# Regulatory role of TRIM21 in the type-I interferon pathway in Japanese encephalitis virus-infected human microglial cells

**DOI:** 10.1186/1742-2094-11-24

**Published:** 2014-02-01

**Authors:** Gunjan Dhawan Manocha, Ritu Mishra, Nikhil Sharma, Kanhaiya Lal Kumawat, Anirban Basu, Sunit K Singh

**Affiliations:** 1 Laboratory of Neurovirology and Inflammation Biology, CSIR-Centre for Cellular and Molecular Biology (CCMB), New R&D Building-1st Floor, Uppal Road, Hyderabad 500007, India; 2 National Brain Research Center (NBRC), Manesar, Haryana, India

**Keywords:** Japanese encephalitis virus, Viral encephalitis, Flavivirus, Antiviral mechanism, Immune evasion, TRIM proteins, TRIM21, Type I interferons, IRF-3, Vector borne infection

## Abstract

**Background:**

Japanese encephalitis virus (JEV) infection leads to Japanese encephalitis (JE) in humans. JEV is transmitted through mosquitoes and maintained in a zoonotic cycle. This cycle involves pigs as the major reservoir, water birds as carriers and mosquitoes as vectors. JEV invasion into the central nervous system (CNS) may occur via antipodal transport of virions or through the vascular endothelial cells. Microglial cells get activated in response to pathogenic insults. JEV infection induces the innate immune response and triggers the production of type I interferons. The signaling pathway of type I interferon production is regulated by a number of molecules. TRIM proteins are known to regulate the expression of interferons; however, the involvement of TRIM genes and their underlying mechanism during JEV infection are not known.

**Methods:**

Human microglial cells (CHME3) were infected with JEV to understand the role of TRIM21 in JEV infection and its effect on type I interferon (IFN-β) production. Cells were infected in presence and absence of exogenous TRIM21 as well as after knocking down the TRIM21 mRNA. Levels of activated IRF3 expression were measured through Western blot analyses of anti-p-IRF3 antibody, and IFN-β production was measured by using IFN-β real-time PCR and luciferase activity analyses.

**Results:**

JEV infection increased expression of TRIM21 in CHME3 cells. JEV induced an innate immune response by increasing production of IFN-β via IRF3 activation and phosphorylation. Overexpression of TRIM21 resulted in downregulation of p-IRF3 and IFN-β, while silencing led to increased production of p-IRF3 and IFN-β in JEV-infected CHME3 cells.

**Conclusion:**

This report demonstrates TRIM21 as a negative regulator of interferon-β (IFN-β) production mediated by IRF-3 during JEV infection in human microglial cells.

## Background

Japanese encephalitis virus (JEV), a flavivirus with single-stranded RNA, is the leading cause of viral encephalitis in most of southeast Asian countries. JEV is transmitted through mosquitoes and maintained in a zoonotic cycle. This cycle involves pigs as the major reservoir/amplifying host, water birds as carriers and mosquitoes as vectors [1]. The estimated worldwide annual incidence of Japanese encephalitis (JE) is about 45,000 human cases and 10,000 deaths [2]. JE leads to long-term neurological damage and significant mortality among children. Approximately 25% of encephalitis patients die, while about 50% of the survivors develop permanent neurologic and/or psychiatric sequelae [1].

The flaviviruses are known to induce proinflammatory response in CNS after infection.

A key step toward induction of innate immunity against viral infections, including JEV, is the production of type I interferons. The presence of virus is sensed by pattern recognition receptors (PRRs) such as Toll-like receptors (TLRs) and RIG-I (retinoic acid-inducible gene 1)-like receptors (RLRs) [3,4]. The engagement of these (receptors) through pathogen molecular patterns can lead to the production of various cytokines and chemokines and other proinflammatory factors. The key regulators of the induction of type I IFNs during viral infections are RIG-I and MDA5 (melanoma differentiation-associated protein 5) [5-10]. These are known to interact with MAVS (mitochondrial antiviral signaling protein), which leads to downstream activation of various kinases such as TBK1/IKKε (TANK-binding kinase 1/I kappa B Kinase-ε), which in turn lead to phosphorylation and activation of various transcription factors to induce IFN-β and IFN-α [11-13]. The production of type I interferons is crucial for generating antiviral response against viruses. Production of interferons is mediated by various transcription factors such as interferon regulatory factors (IRF). Among the IRF family members, IRF-3 has been well documented to play a role in expression of type I interferons in response to viral infections. Phosphorylation of IRF-3 leads to activation, dimerization and nuclear translocation, ultimately leading to the transcription and production of IFN-β. IFN-β further initiates a cascade of signaling events mediated by IRF-7 and IRF-5 resulting in the production of IFN-γ and activation of various interferon-stimulated genes (ISGs) [8,14].

The TRIM family (tripartite-motif family) of proteins has been reported for their roles in regulating the innate immune response to viral infections [15]. TRIM proteins are structurally characterized by a RING domain, a B-box domain and a coiled-coil domain [16,17]. Functionally, most TRIMs are E3 ubiquitin ligases, where RING domains have ubiquitin ligase activity, while the b-Box domains have interacting motifs. TRIM proteins have been reported for their roles in cellular processes such as cell differentiation, transcriptional regulation, signaling cascades and apoptosis [15,18,19]. Many TRIM proteins play important roles in antiviral activities [20]. TRIM5 and TRIM22 are known to restrict HIV replication, while TRIM19 has been reported to restrict VSV and herpes simplex virus (HSV) replication [21-24]. TRIM21 has been known to play a crucial role in regulating type I interferon production, but its role during viral infections is not well understood [25,26]. TRIM21 interacts and ubiquitinates IRF-3, IRF-7 and IRF-8 [27]. Due to such interactions, TRIM21 has been implicated in regulating type I interferon signaling directly by modulating the upstream transcription factors. TRIM21 is part of the RoSSA ribonucleoprotein, which includes a single polypeptide and one of four small RNA molecules. TRIM21 has been reported to recognize and degrade viruses in the cytoplasm by binding to antibody-coated virions [28].

This is the first report showing the role of TRIM21 in modulating the type I interferon response upon JEV infection in human microglial cells. We have demonstrated that induction of TRIM21 during JEV infection is a compensatory mechanism to downregulate the type I interferon production mediated by IRF-3. TRIM21 overexpression leads to downregulation of JEV-mediated activation of IRF-3 and downstream IFN-β production, whereas silencing of TRIM21 results in facilitation of JEV-mediated activation of IRF-3 and upregulation of IFN-β production. We thereby report the inhibitory role of TRIM21 on IFN-β production during JEV infection in human microglial cells.

## Materials and methods

### Materials

The anti-p-IRF3 (Ser396) antibody (#4947S), anti-IRF3 antibody (#4302S), anti-IRF7 (#4920S) and anti-pIRF7 (#5184S) antibodies were purchased from Cell Signaling Technology (Danvers, MA, USA). Anti-β tubulin antibody (#Ab6046) was from Abcam (Cambridge, MA, USA), while anti-TRIM21 antibody (Ro52/SSA) (#sc-25351) was purchased from Santa Cruz Biotechnology (Santa Cruz, CA, USA). Dulbecco’s Modified Eagle’s Medium (DMEM) (#12100–046) was purchased from Gibco (Rockville, MD, USA). Transfection reagents, Lipofectamine 2000 (#1168-019) and GeneCellin (#GC1000) were from Invitrogen (Carlsbad, CA, USA) and BioCellChallenge, respectively. SiRNA against TRIM21 was purchased from Origene (Rockville, MD, USA) along with Negative control scrambled RNA (TRIM21 Trilencer-27 Human siRNA; #SR304594). IFN-β-luciferase promoter was a kind gift from Dr. Adolfo Garcia-Sastre (Mount Sinai School of Medicine, New York City, NY, USA) while JEV (genotype 1 strain # JaOAr) was gifted by Dr. Anirban Basu (NBRC, Manesar, India). Real-time PCR primers were obtained from Bioserve (Hyderabad, India) and IDT (Belgium, Europe).

### Cell culture

Human microglial cell line (CHME3 cells), porcine stable kidney cell line (PS) (for plaque assay) and C6/36 cell line (for viral propagation) were cultured in DMEM (Invitrogen) containing 10% heat-inactivated fetal bovine serum, 100 U penicillin, 100 g/ml streptomycin (#15140122; Invitrogen, Carlsbad, CA, USA) and L-glutamine. All cells were grown in humidified atmosphere containing 5% CO_2_ and 95% air at 37°C (CHME3 and PS cells) and 28°C (C6/36 cells).

### JEV propagation, plaque assay and infection

JEV (genotype 1, JaOAr) was a kind gift from Dr. Anirban Basu, NBRC, India. JEV was further propagated in C6/36 cells. Briefly, C6/36 cells were infected with JEV (MOI 2) in 75 cm^2^ (T-75) cell culture flasks and incubated for 7 days. Post infection, supernatant was collected and precipitated using a PEG viral precipitation kit (#ab102538; Abcam, Cambridge, MA, USA). Plaque-forming units (PFU) of the propagated virus were determined by plaque assay by using porcine stable kidney cells (PS). Cells were seeded at a density of 1.6 × 10^5^ cells in six-well plates and infected with JEV at different dilutions ranging from 10^-3^ - 10^-10^ for 2 h. Post infection, cells were washed with PBS, and 2% low melting agarose overlay [containing 2X-DMEM, 5% FBS and 1% antibiotic (penicillin-streptomycin)] was added to each well. Plates were then incubated at 37°C, 5% CO_2_, for 96 h. Viral plaques were fixed with 10% HCHO and stained with crystal violet in order to count the number of plaques and determine the PFU for JEV. Further infection experiments in this study were performed using JEV at a multiplicity of infection (MOI) of 5 for 24 h, unless otherwise noted. For infection experiments, CHME3 cells were infected with JEV at an MOI of 5 in incomplete DMEM alone for 3 h. Incomplete media were replaced by complete growth media (DMEM with 10% FBS and antibiotics) 3 h later. Cells were harvested at 24 h post infection for RNA and/or protein isolation.

### TRIM21 overexpression and knockdown

**Figure 1:**
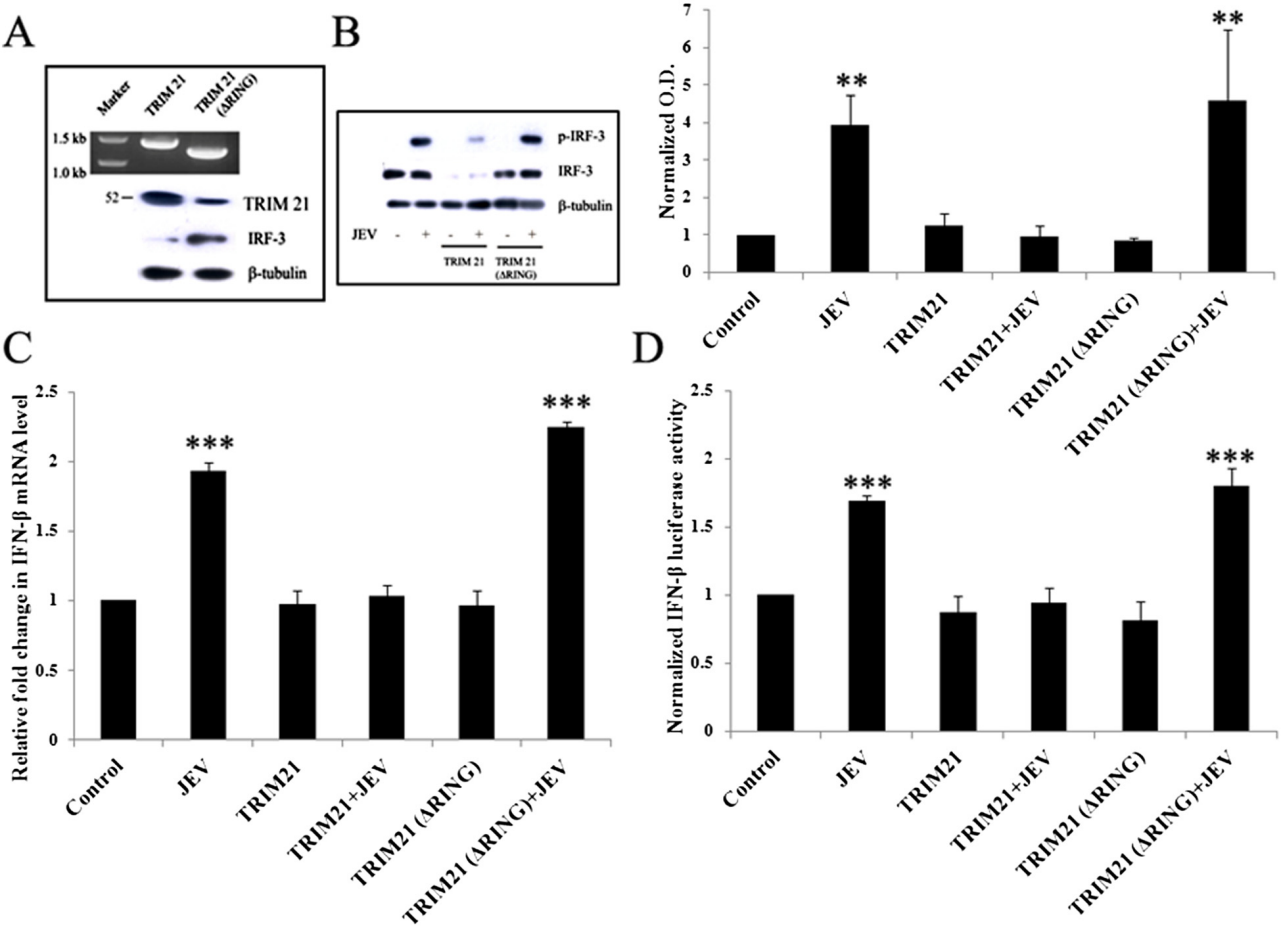
**TRIM21 attenuates the JEV mediated upregulation of the p-IRF3 level and IFN-β level in human microglial cells. (A)** PCR amplification of TRIM21 and TRIM21 (ΔRING) primers was carried out and the product run on 1% agarose gel (*upper panel*). Expression of wild-type TRIM21 as well as the TRIM21 (ΔRING) domain was confirmed by Western blotting (*lower panel*). **(B)** CHME3 cells were transfected with 4 μg of TRIM21 plasmid or TRIM21 (ΔRING) for 48 h. Cell lysates were resolved on *SDS-*PAGE and probed with anti-TRIM21, anti-IRF-3 and anti-β-tubulin antibodies by Western blotting. Representative image is shown. **(C)** Cells transfected with TRIM21 or TRIM21 (ΔRING) were infected with JEV, and total RNA was isolated post 48 h of transfection. Real-time PCR for *IFN-β1* was performed, and an average of three independent sets of experiments is plotted and shown. **(D)** Luciferase assay for IFN-β for cells transfected with TRIM21 or TRIM21 (ΔRING) and infected with JEV along with respective controls was performed. Luciferase activity normalized against β-gal activity was averaged and plotted (*p <0.05, **p < 0.01, ***p < 0.001 from control).

TRIM21 was cloned into the pcDNA3.1 vector. The product was sequenced for confirmation and transformed into DH5α competent cells. The plasmid was then isolated using Qiagen maxi-prep kit (#12163; Qiagen, Hilden, Germany) and observed on a 0.8% agarose gel for purity. A truncated form of TRIM21 without the N-terminal RING domain was also cloned into the pcDNA3.1 vector, to be known as TRIM21 (ΔRING) in this study. TRIM21 protein is comprised of a RING domain from the 16th - 54th amino acid. Therefore, the TRIM21 (ΔRING) primers were designed in such a way that the reverse primer was similar to the wild-type TRIM21 clone, while the forward primer was designed such as to start the polymerase reaction from the 163rd nucleotide so that the RING domain from the wild-type TRIM21 sequence was completely removed. The PCR product was checked on an agarose gel (Figure 1A). The PCR product was further eluted out of agarose gel and digested using EcoR1 and HINDIII enzymes. Along with this insert, the pcDNA3.1 vector was also digested using the same enzymes. Following digestion, the vector and insert were ligated using T4 ligase at 15°C overnight. The ligated product was transformed into *E. coli* DH5α competent cells and spread on an LA agar plate with ampicillin and incubated at 37°C for 15–16 h. The resultant colonies were checked with PCR using TRIM21 (ΔRING) primers, and the positive colonies were further inoculated in LB media to isolate the plasmid using the Qiagen miniprep kit (#27104, Qiagen, Hilden, Germany) according to manufacturer’s protocol. The resultant plasmid was validated by sequencing and used for transfection studies. To validate the expression of truncated TRIM21, CHME3 cells were transfected with TRIM21 (WT) and TRIM21 (ΔRING) for 48 h. Lysates were resolved on SDS-PAGE and probed with for anti-TRIM21 antibody by Western blot. For overexpression, 1 day prior to transfection, CHME3 cells were seeded in 25 cm^2^ cell culture flasks at 70% confluency. Cells were transfected with 4 μg of either TRIM21 or TRIM21 (ΔRING) plasmid per 600,000 cells using GeneCellin transfection reagent in 4 ml transfection media (DMEM with 10% FBS). Cells were replenished with complete growth media (DMEM + 10% FBS + antibiotics) after 8 h of transfection and incubated for 48 h. In cases of infection experiments, 24 h post transfection, cells were infected with JEV at MOI 5 and lysed 24 h post infection. In case of silencing of TRIM21, siRNA against TRIM21 was transfected to CHME3 cells in six-well plates (1.5 × 10^5^ cells per well) using Lipofectamine 2000 transfection reagent. Cells were either non-transfected (control), transfected with scrambled RNA provided in the siRNA kit (negative control) or transfected with siRNA duplex for 48 h. Cells were infected 24 h post transfection in the experiments wherever required.

### Western blotting

Cells were transfected with TRIM21 plasmid for overexpression studies and siRNA for silencing studies as described above. After 48 h of transfection, cells were lysed using RIPA (150 mM NaCl, 50 mM Tris–HCl, pH 7.5, 1% NP-40, 0.5% sodium deoxycholate, 0.1% SDS) buffer containing 1 μM PMSF and 1X protease inhibitor cocktail, ProteCEASE-50 (#427P; G-Biosciences, St. Louis, MO, USA). Protein was quantified using Bradford assay [29]. Lysates were resolved on SDS-PAGE and Western blotted using the desired antibodies against p-IRF3 (1:3,000 dilution), IRF3 (1:3,000), TRIM21 (1:5,000), p-IRF7 (1:3,000) and IRF7 (1:3,000). Optical densities for p-IRF3, p-IRF7 and TRIM21 from visualized Western blots were normalized to their respective loading controls (IRF3/IRF7/β-tubulin) and averaged from three independent experiments.

### Real-time PCR

For RNA isolation, cells were harvested and total RNA isolated using Qiagen RNeasy kit (#74106; Qiagen, Hilden, Germany). Synthesis of cDNA was performed using Superscript II reverse transcriptase system (#11904-018; Invitrogen, Carlsbad, CA, USA) according to manufacturer’s protocol using 2,000 ng RNA. Thermal cycles of cDNA synthesis using random hexamers were as follows: 65°C (5 min); 25°C (10 min); 42°C (50 min); 70°C (10 min); followed by treatment with RNase H for 20 min at 37°C. For amplification, 100 ng cDNA was used as a template for performing RT-PCR using SYBR Green Supermix (#4367659; Applied Biosystems, Warrington, UK). Sequences of all the primers used for various genes in this study are mentioned in Table 1.

**Table 1:** List of primers

**Gene name**	**Primer sequence (5′-3′)**
*TRIM21*	Fwd: 5′ AGAGAGACTTCACCTGTTCTGT 3′
	Rev: 5′ TCAGTTCCCCTAATGCCACCT 3′
TRIM21 (ΔRING)	Fwd: 5′ TACGAATTCCGGCAGCGCTTTCTGCTC 3′
	Rev: 5′ GCCAAGCTTATAGTCAGTGGATCCTTG 3′
*IFN-β1*	Fwd: 5′ GCTCTCCTGTTGTGCTTCTCCAC 3′
	Rev: 5′ CAATAGTCTCATTCCAGCCAGTGC 3′
*β-Actin*	Fwd: 5′ GTCTGCCTTGGTAGTGGATAATG 3′
	Rev: 5′ TCGAGGACGCCCTATCATGG 3′

### IFN-β luciferase assay

CHME3 cells were seeded at a density of 65,000 cells per well in 12-well Plates 1 day prior to transfection. Cells were transfected with IFN-β-Luc reporter plasmid (1 μg/ml) and β-gal (350 ng/ml) in 1 ml of transfection media (DMEM + 10% FBS). In case of TRIM21 overexpression, 1 μg/ml TRIM21 plasmid was co-transfected, while in case of knockdown, 10nM siRNA for TRIM21 was co-transfected. In conditions where infection was required, cells were infected with JEV (MOI 5) 24 h post transfection. After 48 h of transfection (24 h after infection), cells were harvested and luciferase assay performed using the Luciferase assay kit (#E4030; Promega, Madison, WI, USA) according to the manufacturer’s protocol. Cells were lysed in 150 μl 1X lysis buffer and centrifuged at 12,000 g for 3 min, and the supernatant was used for luminescence measurement using the Luciferase assay reagent. Normalization was done by performing a β-galactosidase assay using the β-galactosidase kit (#E2000; Promega, Madison, WI, USA). For this assay, 50 μl of the lysate supernatant was incubated with 50 μl 2X assay buffer at 37°C for 30 min, and the resulting yellow color was read at 420 nm. β-gal activity (in milliunits) in each of the lysates was obtained using a standard curve, and IFN-β luciferase activity was normalized (against β-gal activity), averaged and plotted for three independent sets of experiments.

### Statistical analysis

Data are presented as mean ± standard error. Statistical significance was calculated by using one-way ANOVA and/or Student’s *t*-test. The Tukey-Kramer multiple comparisons post hoc test was used to determine *P*-values.

## Results

### JEV induces IFN-β production in a time-dependent manner in human microglial cells

**Figure 2:**
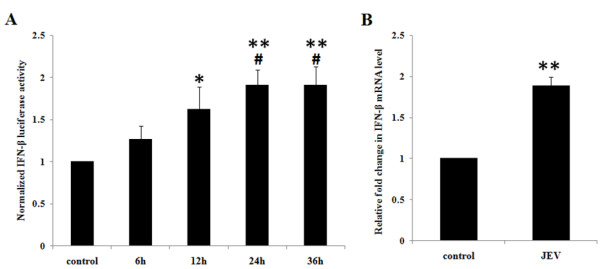
**JEV infection leads to a time-dependent increase in IFN-β luciferase activity in human microglial cells. (A)** CHME3 cells were transfected with IFN-β-luciferase promoter plasmid and β-gal promoter plasmid and infected with JEV at MOI 5 for 6, 12, 24 and 36 h. Luciferase activity was measured, normalized and averaged for three independent experiments. **(B)** CHME3 cells were infected with JEV at MOI 5 for 24 h and total RNA isolated and quantified by real-time PCR for *IFN-β1* levels. Fold change of the *IFN-β1* mRNA level for three independent experiments was averaged and plotted (*p < 0.05, **p < 0.01, ***p < 0.001 from control, ^#^*p* from 6 h, ^$^*p* from 12 h).

In order to verify the innate immune response being generated during JEV infection, we studied the level of IFN-β mRNA as well as IFN-β luciferase activity in microglial cells. Human microglial cells (CHME3) were infected with JEV at an MOI of 5 and harvested at various time intervals of 6, 12, 24 and 36 h. Infected cells were lysed using the reporter lysis buffer for the IFN-β-luciferase assay. IFN-β luciferase activity increased with JEV infection in a time-dependent manner until 24 h (Figure 2A). Since the maximal increase in IFN-β levels was observed at 24 h, all further experiments were performed at the 24-h time point. CHME3 cells were infected with JEV (MOI 5) for 24 h and harvested for RNA isolation. As seen in Figure 2B, IFN-β mRNA levels were significantly increased with JEV infection over the control (Figure 2B). These data confirm the activation of an immune response in terms of IFN-β production following JEV infection in CHME3 cells.

### JEV induces phosphorylation of interferon regulatory factor-3

**Figure 3:**
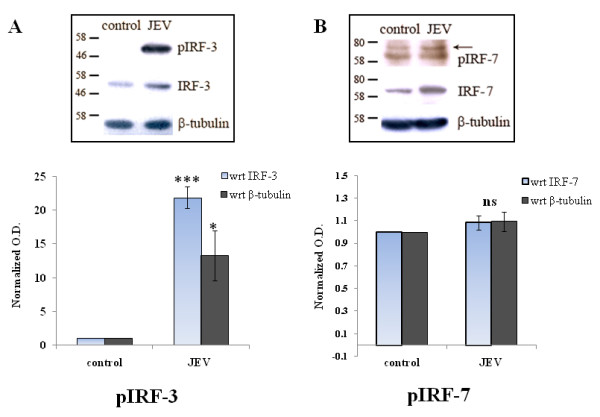
**JEV induces phosphorylated IRF-3 levels in human microglial cells. (A)** CHME3 cells were either un-infected or infected with JEV at MOI 5 for 24 h. Cell lysates were resolved on SDS-PAGE and probed with anti-p-IRF3 antibody, anti-p-IRF7, anti-IRF3 and anti-IRF7 antibodies, and anti-β-tubulin antibody (loading control) by Western blotting. Representatives of three independent experiments are shown. **(B)** Densitometry analyses of Western blot experiments were performed with normalizing p-IRF-3 and p-IRF-7 against their respective controls IRF-3 and IRF-7 as well as against β-tubulin (***p <0.001, *p < 0.05 from control).

In most of the viral infections, the production of type I interferon is a key host immune response. This is mediated by various interferon regulatory factors such as IRF-3 (interferon regulatory factor-3) and IRF-7 (interferon regulatory factor-7). JEV-infected CHME3 cells showed significantly higher levels of active, phosphorylated IRF-3 as compared to controls (Figure 3A). An increase in p-IRF3 levels as seen by Western blotting corresponds with the production of IFN-β (Figure 2), observed at 24 h of JEV infection. Alternatively, IRF-7 phosphorylation was not affected in CHME3 cells infected with JEV at MOI 5 for 24 h (Figure 3B).

### JEV infection induces the expression of TRIM21 in human microglial cells in a time-dependent manner

**Figure 4:**
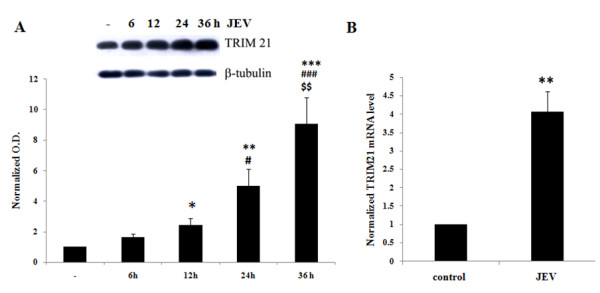
**JEV induces TRIM21 protein levels in human microglial cells in a time-dependent manner. (A)** CHME3 cells were either un-infected or infected with JEV at MOI 5 for 6, 12, 24 and 36 h. Cell lysates were resolved on SDS-PAGE and probed with anti-TRIM21 antibody and anti-β-tubulin antibody (loading control) by Western blotting. A representation of three independent experiments is shown along with densitometry analysis with normalization of TRIM21 against β-tubulin, averaging and plotting. **(B)** CHME3 cells were infected with JEV for 24 h (MOI 5). Total RNA was isolated from harvested cells. cDNA prepared by reverse transcription of control and infected samples was used as a template for qPCR against primers for *TRIM21* gene. Average fold change in the *TRIM21* mRNA level from three independent experiments is plotted and shown (*p < 0.05, **p < 0.01, ***p < 0.001 from control, ^#^*p* from 6 h, ^$^p from 12 h).

TRIM21, an autoantigen, is known to interact with IRF-3, IRF-7 and IRF-8. Because of these interactions, it is a key regulator of the type I interferon immune response. TRIM21 is an E3 ubiquitin ligase that can interact with and ubiquitinate its target molecules. Since JEV induces p-IRF3 and IFN-β expression in human microglial cells (CHME3), we tried to understand the role of TRIM proteins, specifically TRIM21, in regulation of the type I interferon pathway. In order to identify the involvement of TRIM21 in JEV-infected CHME3 cells, we first determined the effect of JEV on the TRIM21 level in the cells. CHME3 cells were infected with JEV (MOI 5) and harvested at various time points. TRIM21 codes for 52 KDa RoSSA protein and Western blotting against the antibody for this protein (from hereon called TRIM21 protein) showed a time-dependent increase in TRIM21 levels for up to 36 h (Figure 4A). CHME3 cells infected with JEV at MOI 5 for 24 h showed increased mRNA levels for the TRIM21 gene (Figure 4B). Additionally, a time-course experiment for JEV infection was performed in HeLa cells to observe the expression of IFN-β as well as TRIM21. Although IFN-β levels increased upon JEV infection after 12 h, there was no change in TRIM21 levels in HeLa cells following JEV infection at any given time point (Additional file 1: Figure S1A & B). This suggested that the role of TRIM21 during JEV infection is specific to human microglial cells.

### TRIM21 overexpression attenuates JEV-mediated upregulation of p-IRF3

Since TRIM21 interacts with IRF-3 directly, it has an important regulatory role in the immune response. We hypothesized that the upregulation of TRIM21 in JEV-infected cells could be a mechanism toward counteracting the production of type I interferons and/or an antiviral response towards the virus infection. The exogenous TRIM21 may further enhance this counteraction and may attenuate the effects in JEV-infected CHME3 cells in terms of IRF3 phosphorylation. Therefore, TRIM21 was overexpressed in CHME3 cells via plasmid transfection in order to observe the effect of TRIM21 on the IRF-3 level and JEV-mediated IRF-3 phosphorylation. The presence of exogenous TRIM21 and TRIM 21 (ΔRING) in CHME3 cells was observed through Western blotting studies. As shown in Figure 1A, TRIM21-transfected CHME3 cells showed TRIM21 antibody reactivity by Western blotting at 52 KDa, while cells transfected with TRIM 21 (ΔRING) showed a band slightly lower than the 52 KDa mark. Cells transfected with TRIM21 were infected by JEV after 24 h of transfection, and lysates were probed for phosphorylated IRF3 levels by Western blotting. As expected, JEV infection increased p-IRF3 levels in CHME3 cells as compared to control (GFP vector-transfected controls). However, overexpression of TRIM21 prior to infection resulted in a reduction of p-IRF3 levels post infection compared to the CHME3 cells only infected with JEV without TRIM21 transfection (Figure 1B). This suggests that TRIM21 could result in attenuation of JEV-mediated IRF-3 activation. Therefore, TRIM21 had an inhibitory effect on IRF3 activation. The total IRF-3 level was unaltered in the CHME3 cells infected with JEV, but TRIM21 overexpression reduced the level of total IRF3. This suggests that TRIM21 directly inhibits unphosphorylated IRF-3 in the presence or absence of JEV infection. This was further validated by using TRIM21 (ΔRING) plasmid in transfection studies. TRIM21 lacking the RING domain had no effect on the IRF3 level (Figure 1A,B). As a result, p-IRF3 levels were also not attenuated in TRIM21 (ΔRING)-transfected CHME3 cells following JEV infection (Figure 1B).

### TRIM21 overexpression represses JEV-mediated elevation in IFN-β levels

We observed an increased production of IFN-β in JEV-infected CHME3 cells (Figure 2). Since TRIM21 transfection attenuates the activation of IRF3, it can be expected that TRIM21 leads to downstream inhibitory effects as well. Hence, in order to understand the role of TRIM21 on IFN-β production, we checked the levels of IFN-β mRNA as well as IFN-β luciferase activity in TRIM21-overexpressed CHME3 cells. Human IFN-β is encoded by the *IFN-β1* gene. CHME3 cells were transfected with TRIM21 plasmid and infected with JEV 24 h post transfection. The CHME3 cells were harvested and RNA isolated for real-time quantification of the *IFN-β1* mRNA level using the primers mentioned in Table 1, and fold change in the mRNA level was determined by normalizing the *IFN-β1* C_t_ values against those of *β-actin*. As observed in Figure 2B, we found an increased expression of *IFN-β1* at mRNA levels in JEV-infected CHME3 cells as compared to vector controls (Figure 1C). However, we observed the reduction in *IFN-β1* levels in JEV-infected TRIM21-transfected CHME3 cells in accordance with IRF-3 activation compared to controls. CHME3 cells transfected with truncated TRIM21 lacking the RING domain showed no reduction in *IFN-β1* mRNA levels. A similar trend was observed with IFN-β luciferase activity. Cells were transfected with IFN-β-luciferase promoter and β-gal with or without TRIM21 plasmid followed by JEV infection. Luciferase activity was measured 48 h post transfection. JEV-infected cells showed significantly higher luciferase activity of IFN-β-luciferase promoter; however, transfection of TRIM21 before JEV infection attenuated JEV-mediated luciferase activity (Figure 1D). This observation suggests that TRIM21 has an inhibitory role in interferon-β production after JEV infection.

### TRIM21 silencing facilitates JEV-mediated upregulation of the p-IRF3 level in CHME3 cells

**Figure 5:**
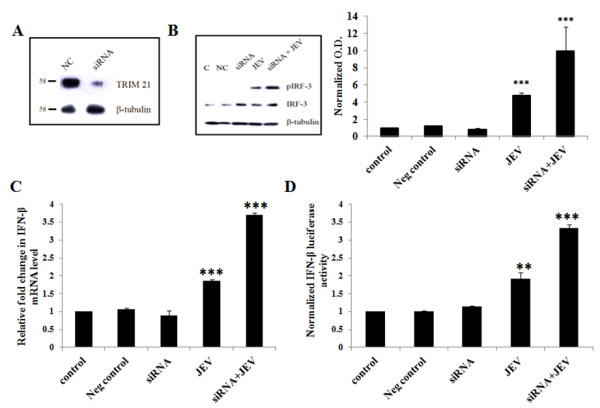
**TRIM21 knockdown facilitates JEV-mediated IRF3 activation and upregulation of the IFN-β level. (A)** Cells were either transfected with negative control RNA (*NC*) or transfected with 10nM siRNA against TRIM21 for 48 h. Cell lysates were resolved on SDS-PAGE and probed with anti-TRIM21 antibody and anti-β-tubulin antibody by Western blotting. A representative image is shown. **(B)** Cells were either non-transfected (C), transfected with negative control RNA (NC) or with TRIM21 siRNA for 24 h followed by JEV infection for 24 h. Cell lysates were resolved on SDS-PAGE and probed with anti-p-IRF3 antibody, anti-IRF3 and anti-β-tubulin antibodies (loading control) by Western blotting. A representative of three independent experiments is shown. Densitometry analyses of Western blot experiments were performed with normalizing p-IRF-3 and p-IRF-3 against β-tubulin. **(C)** Real-time PCR for *IFN-β1* for siRNA-transfected and JEV-infected cells along with the respective controls was performed and averaged for three independent sets of experiments. **(D)** Luciferase assay for IFN-β for cells transfected with siRNA against TRIM21 and infected with JEV along with respective controls was performed. Luciferase activity normalized against β-gal activity was averaged and plotted (**p < 0.01, ***p < 0.001 from control).

To further confirm the inhibitory role of TRIM21 on IFN-β production, siRNA against TRIM21 was used to knock down the expression of TRIM21 in CHME3 cells. Silencing of TRIM21 was performed by using commercially available siRNA duplexes against TRIM21 that resulted in >70% knockdown of TRIM21 as shown in Figure 5A. Since JEV infection stimulated IRF3 activation and phosphorylation, this phenomenon was checked following TRIM21 silencing. JEV-infected CHME3 cells showed increased phosphorylation of IRF3 as compared to controls; however, silencing of TRIM21 prior to infection further enhanced the phosphorylation and activation of IRF3 (Figure 5B). This further confirmed the inhibitory role of dysregulated endogenous TRIM21 during JEV infection in human microglial cells.

### TRIM21 silencing upregulates the JEV-mediated increase in IFN-β level

Further downstream from IRF3 activation, the expression level of IFN-β was checked in order to confirm the role of TRIM21 in modulating the expression of the interferon in case of JEV infection. Again, corresponding to the IRF3 activation, JEV-infection resulted in an additional increase in the IFN-β mRNA level as well as IFN-β-luciferase activity in CHME3 cells, where TRIM21 was silenced prior to the JEV infection (Figure 5C,D).

## Discussion

This study reports that JEV infection induces the expression of TRIM21 in human microglial cells. Exogenous overexpression of TRIM21 attenuates JEV-mediated activation and phosphorylation of IRF3 as well as expression of IFN-β levels. In addition, TRIM21 silencing leads to facilitation of JEV-mediated IRF3 phosphorylation and downstream induction of IFN-β.

We demonstrated that JEV induces IRF3 activation at 24 h of JEV infection. However, levels of phosphorylated IRF7 are not significantly different in the infected cells as compared to control after 24 h. IRF7 has been reported to become activated only during the early phases of JEV infection, and the levels of p-IRF7 are not elevated during late phases of JEV infection [30]. Therefore, our study was focused on understanding the role of TRIM protein during JEV infection in the type I interferon pathway in terms of IRF3 activation as well as its effect on the expression of IFN-β. The production of IFN-β by JEV is known to occur through the RIG-1-mediated pathway [3]. However, the role of TRIM proteins in modulating the immune response in JEV infection had not yet been reported.

TRIM proteins are known to act as antiviral molecules because of their specific actions against specific viruses, as well as regulators of immune signaling pathway [15,25,31,32]. TRIM79α is reported to restrict viral replication of the tick-borne encephalitis virus (TBEV), but not other flaviviruses, such as West Nile virus [33]. TRIM56 has been reported to restrict bovine viral diarrhea virus replication *in vitro*[34]. The antiviral activity of TRIM21 has been reported to be through the antibody-mediated pathway [28,35]. It targets the virus toward proteasomal degradation by ubiquitination. Vaysburd *et al.*[28] showed that sensing of antibody-coated pathogens through TRIM21 increased in the case of nonenveloped DNA viruses, RNA viruses and intracellular bacterial infections [28,35]. While TRIM21 acts as an Fc receptor in sensing the antibody-coated viruses, the modulation of the immune signaling pathway by TRIM21 is dependent on the targeting of interferon regulatory factors via ubiquitination. We observed that JEV increases the expression of TRIM21 in CHME3 cells as a negative regulator of the type I interferon-mediated pathway. Two schools of thoughts exist regarding the action of TRIM21 in modulating the interferon pathway [25-27,36-38]. One suggests that TRIM21 promotes degradation of IRF-3 and IRF-7, thereby limiting type I interferon production. Our study supports the same findings as we observed the degradation of IRF-3 by TRIM21 in CHME3 cells; hence, the availability of less phosphorylated forms of IRF-3 ultimately leads to reduced expression of IFN-β [26,37]. Since there was no change in IRF3 levels and subsequent IFN-β levels upon TRIM21 (ΔRING) transfection, it suggested that the E3 ligase activity of TRIM21 may be responsible for the regulation of IRF3 expression. Our finding supports the those of Higgs *et al.*[26] in which they showed that both the RING domain and the SPRY domain are equally required to regulate the expression of IRF3 by the interaction and proteasomal degradation of IRF3 [26]. Hence, the absence of any one of the domains is sufficient to validate the role of TRIM21 in IRF3 and IFN-β regulation during JEV infection. The other school of thought supports the stabilization of IRF3 expression through a TRIM21-mediated pathway. This has been reported in the case of Sendai virus infection, where TRIM21 acts as a positive regulator of the IRF3 pathway during viral infection [27]. This functional variability could be attributed to being dependent on the type of viral infection, the duration of infection and the upstream signaling pathway. Apart from RNA virus recognition, TRIM21 is also able to modulate DNA virus infections. TRIM21 has been shown to interact with DDX41, a cytosolic DNA sensor that triggers type I IFN responses [39]. TRIM21 causes ubiquitination of DDX41, leading to a lesser production of IFN-β. A number of other TRIM proteins are also involved in modulating the inflammatory signaling pathways. TRIM30 (TRIM79α) is reported to be involved in lysosomal degradation of TAK1 binding protein 2 (TAB2) and TAK1 binding protein 3 (TAB3) downstream of the TLR4 pathway, leading to the inhibition of NF-ҡB induction during LPS stimulation [40]. Like TRIM21, TRIM27 is also known to target IKKs, thereby negatively regulating PRR pathways [41]. TRIM5α is also known to negatively regulate NF-ҡB and mitogen-activated protein kinase (MAPK) signaling pathways by targeting TGF-β-activated kinase 1 (TAK1), and these activities are known to be uncoupled from the retroviral capsid recognition [42,43]. TAK1 has also been reported to be targeted by TRIM8 [44]. TRIM5α has also been reported to positively regulate NF-ҡB signaling, AP-1 activation and expression of proinflammatory cytokines [42]. TRIM23 acts as a cofactor in the regulation of NF-ҡB activation in human cytomegalovirus infection [45]. Therefore, the modulation of immune responses by TRIM proteins can be highly specific based on the type of pathogens and their derivatives.

**Figure 6:**
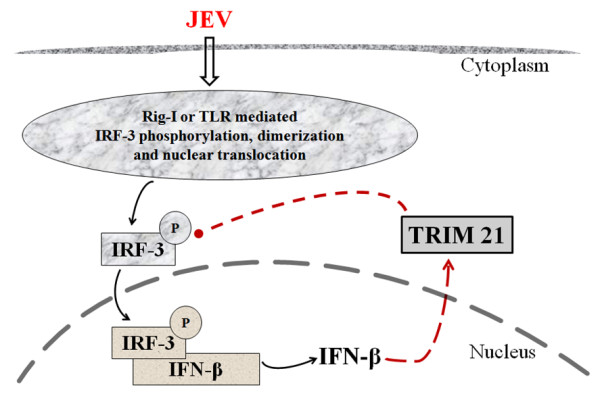
**Model showing a plausible role of TRIM21 as a negative regulator of IRF3 activation and IFN-β production following JEV infection in human microglial cells.** JEV infection causes activation of the RIG-1 receptor, initiating a downstream signaling mechanism leading to the activation of IRF-3. Phosphorylated IRF-3 dimerizes and translocates into the nucleus, where it leads to the transcription and production of IFN-β. JEV infection also induces the TRIM21 protein, which negatively regulates IRF-3 phosphorylation, leading to reduced IFN-β production. The upregulation of TRIM21 is proposed to be a feedback mechanism to inhibit the innate immune response in JEV infection.

The increased expression of TRIM21 during JEV infection and its inhibitory role act as a feedback mechanism to attenuate the immune response (Figure 6). This is supported by the observation that the activation of IRF3 in JEV infection is further enhanced by knockdown of TRIM21 in CHME3 cells prior to infection. Consequently, the IFN-β level was also increased in JEV-infected TRIM21 knocked-down cells. The expression of TRIM21 has also been reported to be induced by IFN-α and IFN-β via the IRF-mediated pathway [46]. Other TRIM proteins, such as TRIM5α and TRIM19, can also be altered by type I IFNs [47-51]. Two independent studies have reported the expression of many TRIM proteins following type I interferon stimulation in mouse macrophages, dendritic cells, primary human-monocyte-derived macrophages and human primary lymphocytes [47,48]. Zhao *et al.*[52] reported that TRIM 38 acts as a negative feedback regulator of NF-ҡB signaling in TLR agonist-treated macrophages [52]. This evidence along with our observations suggests the involvement of a possible feedback mechanism for the expression of TRIM proteins by interferon-mediated responses during viral infections. In summary, we suggest that JEV infection in human microglial cells triggers an innate immune response in terms of IFN-β production. The expression of IFN-β in turn is suppressed by increased expression of TRIM21 during JEV infection in human microglial cells. Probably JEV utilizes this strategy to suppress the type I interferon response as a part of an immune evasion mechanism. However, further studies are required to understand the involvement of other TRIM proteins as an antiviral factor against other flaviviruses in order to understand the mechanisms of pathogenesis as well as to develop therapeutic tools.

## Conclusion

JEV infection in human microglial cells induced the expression of TRIM21 protein. Exogenous expression of TRIM21 in human microglial cells resulted in attenuation of JEV-mediated effects in terms of activation of interferon regulatory factor-3 and production of interferon-β. Further knockdown of TRIM21 enhances the JEV-mediated IRF-3 activation and IFN-β production. Although upstream mechanisms of JEV-mediated IRF-3 phosphorylation are known, this study provides the first evidence of the involvement of TRIM21 protein in negatively regulating the innate immune response by targeting IRF-3-mediated IFN-β production during JEV infection. JEV could exploit this strategy to suppress the type I interferon response during the early course of infection.

## Abbreviations

JEV: Japanese encephalitis virus; JE: Japanese encephalitis; PRR: Pattern recognition receptors; RLR: RIG-1 like receptors; RIG-1: Retinoic acid-inducible gene 1; MDA5: Melanoma differentiation-associated protein 5; MAVS: Mitochondrial antiviral signaling protein; TLR: Toll-like receptors; TBK: Tank binding kinase; TGF: Transforming growth factor; IFN: Interferon; IRF: Interferon regulatory factor; TRIM: Tripartite motif; ISG: Interferon-stimulated genes; TAB2: TAK1 binding protein 2; TAB3: TAK1 binding protein 3; LPS: Lipopolysaccharide; MAPK: Mitogen-activated protein kinase; HSV: Herpes simplex virus.

## Competing interests

The authors declare that they have no competing interests.

## Authors’ contributions

GDM designed the study, performed experiments and data analysis, and cowrote the manuscript. RM performed the PCR of the TRIM customized plate. NS carried out cloning work of TRIM21 and TRIM21 (ΔRING); KLM propagated the JEV in mice. AB provided the CHME3 cells and JEV as a kind gift. SKS conceived the idea, supervised the experiments and data analysis, and wrote the manuscript. All authors have read and approved the final version of the manuscript.

## Supplementary Material

Additional file 1: Figure S1 JEV infection does not affect TRIM21 protein levels in HeLa cells. HeLa cells were infected with JEV at MOI 5 and harvested at different time intervals of 6, 12, 24 and 36 h. Cells were either lysed for Western blotting against anti- TRIM21 antibody **(A)** or lysed using reporter lysis buffer for IFN-β luciferase assay **(B)**. All experiments were performed as sets of three independent experiments and data averaged and plotted as mean ± SEM (*p < 0.05, **p < 0.01, ***p < 0.001 from control, ^#^p from 6 h, ^$^p from 12 h). Click here for file
