# Supplementary figures and images for: Regulatory role of TRIM21 in the type-I interferon pathway in Japanese encephalitis virus-infected human microglial cells
Source: J Neuroinflammation. 2014 Feb 1;11:24. doi: 10.1186/1742-2094-11-24 (PMC3922089; doi:10.1186/1742-2094-11-24)

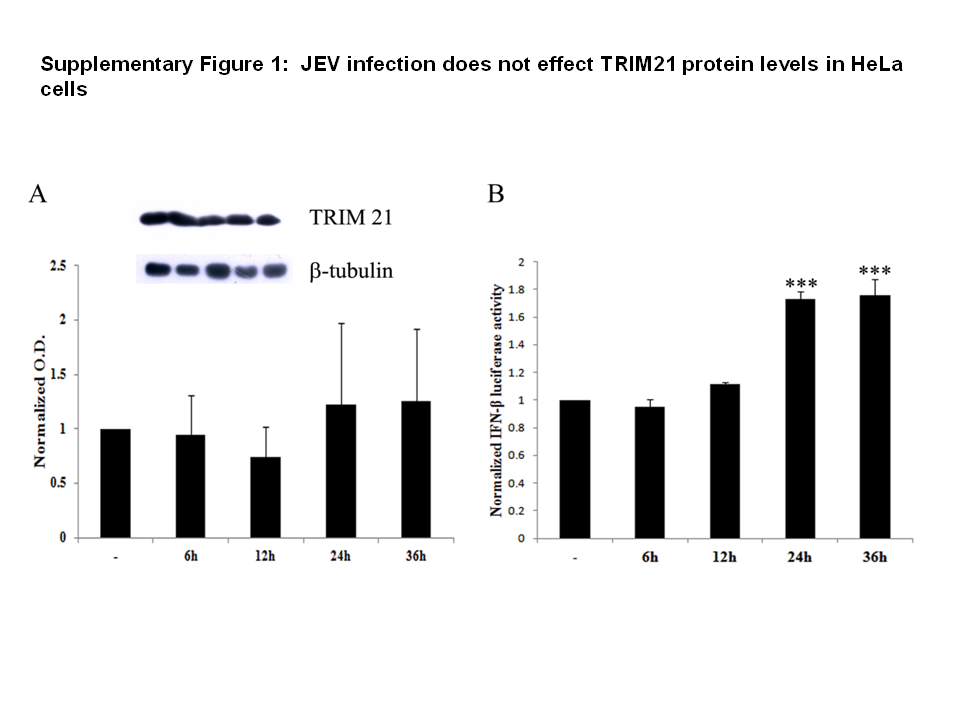

Supplement: Additional file 1: Figure S1 — JEV infection does not affect TRIM21 protein levels in HeLa cells. HeLa cells were infected with JEV at MOI 5 and harvested at different time intervals of 6, 12, 24 and 36 h. Cells were either lysed for Western blotting against anti- TRIM21 antibody (A) or lysed using reporter lysis buffer for IFN-β luciferase assay (B). All experiments were performed as sets of three independent experiments and data averaged and plotted as mean ± SEM (*p < 0.05, **p < 0.01, ***p < 0.001 from control, #p from 6 h, $p from 12 h). [file 1742-2094-11-24-S1.tiff]
